# Selection for Plastic, Pathogen-Inducible Recombination in a Red Queen Model with Diploid Antagonists

**DOI:** 10.3390/pathogens10070898

**Published:** 2021-07-15

**Authors:** Sviatoslav Rybnikov, Zeev Frenkel, Abraham B. Korol, Tzion Fahima

**Affiliations:** 1 Institute of Evolution, University of Haifa, 199 Abba-Hushi Avenue, Haifa 3498838, Israel; sviatoslav.rybnikov@gmail.com (S.R.); zvfrenkel@gmail.com (Z.F.); 2 Department of Evolutionary and Environmental Biology, University of Haifa, 199 Abba-Hushi Avenue, Haifa 3498838, Israel

**Keywords:** host-parasite co-evolution, recombination plasticity, matching-phenotype interaction, diploid selection, modifier model

## Abstract

Antagonistic interactions and co-evolution between a host and its parasite are known to cause oscillations in the population genetic structure of both species (Red Queen dynamics). Potentially, such oscillations may select for increased sex and recombination in the host, although theoretical models suggest that this happens under rather restricted values of selection intensity, epistasis, and other parameters. Here, we explore a model in which the diploid parasite succeeds to infect the diploid host only if their phenotypes at the interaction-mediating loci match. Whenever regular oscillations emerge in this system, we test whether plastic, pathogen-inducible recombination in the host can be favored over the optimal constant recombination. Two forms of the host recombination dependence on the parasite pressure were considered: either proportionally to the risk of infection (prevention strategy) or upon the fact of infection (remediation strategy). We show that both forms of plastic recombination can be favored, although relatively infrequently (up to 11% of all regimes with regular oscillations, and up to 20% of regimes with obligate parasitism). This happens under either strong overall selection and high recombination rate in the host, or weak overall selection and low recombination rate in the host. In the latter case, the system’s dynamics are considerably more complex. The prevention strategy is favored more often than the remediation one. It is noteworthy that plastic recombination can be favored even when any constant recombination is rejected, making plasticity an evolutionary mechanism for the rescue of host recombination.

## 1. Introduction

Antagonistic interactions, like those between a host and its parasite, are long recognized to cause more or less regular oscillatory dynamics. In the early 1920s, Lotka [1] and Volterra [2] theoretically predicted oscillations in the *ecological* dynamics of the antagonists, i.e., in size/density of their populations. The anticipated pattern was then observed in both field and lab [3,4,5]. Later on, Haldane [6] hypothesized, preceding from general speculations on the negative frequency-dependent selection, that the antagonistic interactions should cause oscillations also in the *genetic* dynamics of the antagonists, i.e., in allele frequencies of their interaction-mediating genes. This phenomenon is now known as the “Red Queen dynamics”, following the colorful terminology introduced by Van Valen [7] and adopted by Bell [8]. It has become widely discussed before empirical tests for its existence [9,10], and even for the existence of negative frequency-dependent selection [11,12,13].

In the late 1970s–early 1980s, several authors speculated, more or less explicitly, that the Red Queen dynamics might favor sex/recombination in the host (see [14] for a historical review). Following Bell [8], this assumption is now referred to as the Red Queen hypothesis. Although the first formal model did confirm it [15], further simulations showed that it is less generic than expected [16,17]. It is clear now that the emergence of selection for sex/recombination under antagonistic interactions depends on various factors: interaction type [18,19], antagonism severity [20], selection intensity, at least in the parasite [21], epidemiological context [22], etc. In general, empirical studies support the Red Queen hypothesis—although the evidence remains rather scarce for sex [23,24,25,26,27,28,29] and even more so for recombination [30,31].

Here, we also examine the evolution of recombination under antagonistic interactions. However, we do not aim to test the Red Queen hypothesis per se. Instead, we focus on a more specific question: whether antagonistic interactions can favor recombination *plasticity*, which is one of the most interesting features of recombination. By recombination plasticity, we refer to the situation when the recombination rate of a given genotype varies across environments. This phenomenon was first reported by Plough more than a century ago, for fruit flies reared under a spectrum of temperature regimes [32,33]. To date, similar results are obtained for many other species exposed to various environmental stressors (for recent reviews, see [34,35]), including biotic stressors caused by interspecific antagonistic interactions [36,37,38,39]. The consistent pattern in all such studies is that the organisms tend to display a higher recombination rate in stressful than benign environmental habitats/periods.

In the current study, the stressor which can potentially induce a plastic response in an organism’s recombination is its virulent pathogen, namely its presence inside the host or its frequency in the external environment. Empirical evidence, although very limited, indicates that pathogens may exert a recombinogenic effect on their hosts [36,37,38,39], but the evolvability of plastic recombination in the host under this scenario has not been modeled yet, to the best of our knowledge. Here, we numerically simulate the co-evolution of the two antagonists under various parameter combinations. Whenever this co-evolution leads to fairly regular oscillations, we test whether plastic, pathogen-inducible recombination in the host can be favored over the optimal constant, pathogen-independent recombination. We consider two forms of pathogen-inducible recombination: (i) with recombination rate increased *before* the interaction, proportionally to the risk of infection (hereafter—“prevention strategy”), and (ii) with recombination rate increased *after* the interaction, based on the fact of infection (hereafter—“remediation strategy”).

Both antagonists in our model are diploids. The focus is made on diploids for several reasons. First, the recombinogenic effect of interspecific antagonistic interactions was reported for diploids [36,37,38,39]. Second, we previously examined the evolution of plastic recombination in diploids under abiotic fluctuations [40,41], which potentially allows us to generalize the results of the current study.

It must be noted that, although recombination is an intrinsic component of canonical meiosis, selection on recombination and selection on sex may occur under different parameter ranges, especially in diploids [42,43]. Until now, antagonistic interactions have been tested only for the evolutionary advantage of plastic sex [44] but not plastic recombination. Hence, here we focus on recombination per se, assuming obligate sex in both antagonists.

## 2. Model and Methods

### 2.1. Life Cycles

Both antagonists have infinite populations. Both reproduce sexually, with non-overlapping generations. In both species, the life cycle includes maturation, meiosis, and random mating. Let $z_{ij}^{t}$ and $a_{ij}^{t}$ be, respectively, the frequencies of zygote and adult of genotype *ij* (*i* and *j* stand for the parental haplotypes) in the current generation *t*. During maturation, the genotypes demonstrate differential viability ( $W_{ij}$ ), so that selection changes the frequency as follows:

(1) $$a_{ij}^{t}=\frac{z_{ij}^{t}\cdot W_{ij}}{\bar{W}}$$

where the denominator stands for the population’s mean fitness:

(2) $$\bar{W}=\sum_{ij}z_{ij}^{t}\cdot W_{ij}$$

The frequency of gamete *k* in the gamete pool, $g_{k}$ , can be calculated as:

(3) $$g_{k}^{t}=\sum_{ij}a_{ij}^{t}\cdot P_{ij\to k}$$

where $P_{ij\to k}$ is the probability to obtain gamete *k* from adult *ij*. Upon random mating, the frequency of each zygote in the next generation is proportional to the frequencies of both parental gametes in the current generation:

(4) $$z_{ij}^{t+1}=g_{i}^{t}\cdot g_{j}^{t}$$

### 2.2. Species Interaction

Each species bears two linked loci that determine its interaction with the antagonist (hereafter—“interaction-mediating loci”): $A^{h}$ and $B^{h}$ in the host, $A^{p}$ and $B^{p}$ in the parasite. Each locus is represented by two possible alleles: $A_{1}/A_{2}$ , $B_{1}/B_{2}$ ; one of them is completely dominant over the other, so the heterozygote displays a phenotype equal to that of one of the two homozygotes. For simplicity, we assumed the same dominance at all interaction-mediating loci *within* each antagonist. However, *between* the antagonists, we allowed both the same and the opposite dominance; these two situations are referred to as “in-phase dominance” and “anti-phase dominance”, respectively.

The encounter between a host and a parasite results in one of the two alternative outcomes: either infection or resistance. If the antagonists differ at least at one selected locus, the host “recognizes” the parasite and develops resistance; otherwise, the parasite succeeds to infect. This scheme can be viewed as a diploid extension of the canonical haploid “matching-genotype” interaction (see [19] for a review of the interaction types) and can be referred to as “matching-phenotype” interaction. Table 1 summarizes the outcomes for both in-phase and anti-phase dominance.

The encounter outcome (infection/resistance) defines finesses of both antagonists, $\omega^{h}$ and $\omega^{p}$ . The host displays a reduced fitness when infected, while the parasite suffers from a loss in fitness when fails to infect:

(5) $$\begin{array}{c} \omega^{h}=\left\{\begin{array}{cc} 1 & \mathrm{resistance}\\ 1-s^{h} & \mathrm{infection} \end{array}\right.\\ \omega^{p}=\left\{\begin{array}{cc} 1-s^{p} & \mathrm{resistance}\\ 1 & \mathrm{infection} \end{array}\right. \end{array}$$

where coefficients $s^{h}$ and $s^{p}$ describe selection intensity in the host and the parasite, respectively.

Both species are subject to frequency-dependent selection: the encounters between host *i* and parasite *j* occur proportionally to their frequencies in the corresponding populations, $h_{i}$ and $p_{j}$ . Thus, the fitness of the whole class, $W_{i}^{h}$ and $W_{j}^{p}$ , can be calculated as the expected values of $\omega_{ij}^{h}$ and $\omega_{ij}^{p}$ :

(6) $$\begin{array}{c} W_{i}^{h}=\sum_{j}\omega_{ij}^{h}\cdot p_{j},\\ W_{j}^{p}=\sum_{i}\omega_{ij}^{p}\cdot h_{i} \end{array}$$

### 2.3. Recombination Strategies

Recombination rate in the parasite $r^{p}$ is fixed and treated as a parameter. Recombination rate in the host $r^{h}$ is determined by genotype at a selectively neutral locus (hereafter—“modifier locus”). The modifier locus is unlinked from the interaction-mediating loci. The interaction between any pair of modifier alleles is purely co-dominant.

The modifier alleles confer alternative recombination strategies: (a) *constant* recombination—with a certain rate equal for all hosts; (b) *plastic*, pathogen-induced recombination —with the rate varying among the hosts. Within plastic recombination, two sub-strategies are considered:

- prevention strategy—with recombination sensitive to the potential infection risk, so that recombination rate of each host class increases proportionally to the frequency p of the dangerous (exactly for this class) parasite class:
(7) $$r^{h}=r_{\min}^{h}+p\cdot \left(r_{\max}^{h}-r_{\min}^{h}\right).$$
- remediation strategy—with recombination sensitive to the actual infection status, so that the infected hosts display an increased recombination rate ( $r_{\max}^{h}$ ) compared to their resistant counterparts ( $r_{\min}^{h}$ ):
(8) $$r^{h}=\left\{\begin{array}{cc} r_{\min}^{h} & \mathrm{resistance}\\ r_{\max}^{h} & \mathrm{infection} \end{array}\right.$$

Both sub-strategies imply a negative association between recombination rate and conditions: poor conditions (either infection or its high risk) lead to a higher recombination rate.

### 2.4. Experimental Design

All recombination strategies are compared in terms of the modifier approach [45,46]. Specifically, to compare two arbitrary recombination strategies, $S_{1}$ and $S_{2}$ , we examined the competition between the corresponding modifier alleles, $M_{1}$ and $M_{2}$ . The system first evolved during 10,000 generations with modifier allele $M_{1}$ . Upon this “burn-in” period, required for the system to establish the oscillation, the competing allele $M_{2}$ was injected in low frequency (0.05) at both within- and between-locus equilibria, and its dynamics were traced over 10,000 additional generations. The same procedure was also repeated from the opposite side: the system first evolved with allele $M_{2}$ , and then the allele $M_{1}$ was introduced in rarity. Based on these two tests, strategy $S_{1}$ was recognized more favorable over strategy $S_{2}$ if allele $M_{1}$ succeeded to spread in the population while allele $M_{2}$ failed to do this. If both alleles succeeded to spread, leading to a stable polymorphism at the modifier locus, the partial advantage was ascribed to each strategy.

For the analysis, we generated a set of 10,000 random parameter combinations, each defined by three parameters: selection intensity in the host ( $s^{h}$ ), selector intensity in the parasite ( $s^{p}$ ), and recombination rate in the parasite ( $r^{p}$ ). The values of $s^{h}$ and $s^{p}$ were distributed uniformly from 0 to 1 while those of $r^{p}$ —from 0 to 0.5. This set was applied to both in-phase and anti-phase dominance, leading to a total of 20,000 regimes analyzed. For each regime, we first assessed the optimal constant recombination rate in the host ( $r_{\mathrm{opt}}^{h}$ )—the rate that was favored over both lower and higher rates. To this end, we used two series of pairwise comparisons: one starting from recombination rate 0 and moving upwards, and the other starting from recombination rate 0.5 and moving downwards. Together, these two series gave a lower and an upper estimate for the sought $r_{\mathrm{opt}}^{h}$ . This procedure was then repeated four times, each with higher accuracy, to obtain a further narrow gap between the two estimates ( ε ).

For further analysis, we focused only on regimes leading to fairly regular oscillations. As an operational criterion for such regularity, we used the accuracy of the estimated $r_{\mathrm{opt}}^{h}$ , by requiring ε≤0.001 . For the chosen regimes, we tested whether each of the two plastic strategies, the prevention and remediation ones, was favored over the found optimal constant strategy. Under plastic strategies, the recombination rate varied around $r_{\mathrm{opt}}^{h}$ with magnitude ∆r : from $r_{\min}^{h}=r_{\mathrm{opt}}^{h}-∆r$ to $r_{\max}^{h}=r_{\mathrm{opt}}^{h}+∆r$ . For regimes where constant recombination was rejected ( $r_{\mathrm{opt}}^{h}$ = 0), recombination rate under plastic strategies varied from $r_{\min}^{h}=0$ to $r_{\max}^{h}=∆r$ . We considered four magnitudes of plasticity: ∆r=0.001 , ∆r=0.0025 , ∆r=0.005 , and ∆r=0.01 .

Additionally, we examined in more detail the case of obligate parasitism, implying that the parasite survives only if it succeeds to infect ( $s^{p}=1$ ). Importantly, since the proportion of selection regimes favoring plastic recombination depends on the chosen parameter range, such percentages should only be compared for the same range (e.g., when alternative plastic strategies are examined).

The code for the simulations is written in GNU Octave 6.1 (Supplementary File S1). The simulations were deployed at the Galileo platform for remote computations. The obtained results were statistically analyzed using JASP 0.14.

## 3. Results

### 3.1. The System’s Dynamics and the Optimal Constant Recombination in the Host

The system’s dynamics considerably depended on the three examined parameters ( $s^{h}$ , $s^{p}$ , and $r^{p}$ ), as well as recombination rate in the host ( $r^{h}$ ). Under weak selection ( $s^{h}$ , $s^{p}<0.1$ ), the oscillations had low amplitude, if they emerged at all. Stronger selection in each of the antagonists led to more pronounced and more regular oscillations. Interestingly, the most complex dynamics was observed under intermediate selection: $s^{h}$ , $s^{p}=0.4\ldots 0.6$ . All other things equal, higher recombination rate in the parasite ( $r^{p}$ ) usually made the dynamics more regular (Supplementary Figure S1).

In its turn, the complexity of oscillations affected the estimation of the optimal constant recombination in the host ( $r_{\mathrm{opt}}^{h}$ ). The used algorithm returned the lower and the upper estimates of $r_{\mathrm{opt}}^{h}$ , and we limited our further analysis only to regimes where $r_{\mathrm{opt}}^{h}$ could be estimated with sufficient accuracy: ε≤0.001 (about one-third of the total tested set of parameter combinations). Moreover, in a considerable proportion of regimes (~20–40%, depending on in-phase vs. anti-phase dominance), there existed two local optima for $r^{h}$ . The oscillations in the vicinity of the lower optimum ( $r_{\mathrm{opt}}^{h}≅0)$ were often rather complex. In contrast, the upper optimum could typically be estimated accurately given the fairly regular oscillations. For such regimes with bistability, only the upper optimum was included in further analysis.

The predominant proportion of regular regimes appeared to favor non-zero recombination in the host. Among these regimes, the values of $r_{\mathrm{opt}}^{h}$ were on average slightly higher under in-phase than anti-phase dominance: ~0.17 against ~0.15. Moreover, in-phase dominance allowed higher maximum values of $r_{\mathrm{opt}}^{h}$ than anti-phase dominance: up to ~0.44 against ~0.33. Only rarely, under very weak selection in at least one of the antagonists, recombination in the host was rejected ( $r_{\mathrm{opt}}^{h}=0)$ .

We then used multivariate linear regression to estimate the relative effects of the three examined parameters ( $s^{h}$ , $s^{p}$ , and $r^{p}$ ) on $r_{\mathrm{opt}}^{h}$ . All three tended to increase $r_{\mathrm{opt}}^{h}$ regardless of dominance. At that, $s^{p}$ and especially $s^{h}$ appeared much more influential than $r^{p}$ (Table 2, Models A). Moreover, to a certain extent, selection intensities in the two antagonists “compensated” one another, so that close values of $r_{\mathrm{opt}}^{h}$ occurred either under low $s^{h}$ and high $s^{p}$ or under high $s^{h}$ and low $s^{p}$ . Regression models allowing parameter interactions confirmed that the overall selection intensity ( $s^{h}\cdot s^{p}$ ) affects $r_{\mathrm{opt}}^{h}$ stronger than any of the two, $s^{h}$ and $s^{p}$ , alone (Table 2, Models B). Yet, the interaction between $s^{h}$ and $s^{p}$ seems more complicated than their product $s^{h}\cdot s^{p}$ , since considering both parameters and their combinations further increased the models’ performance (Table 2, Models C). Overall, the models succeeded to explain ~89% to 96% of the variation in the dependent variable (Table 2).

**Figure 1:**
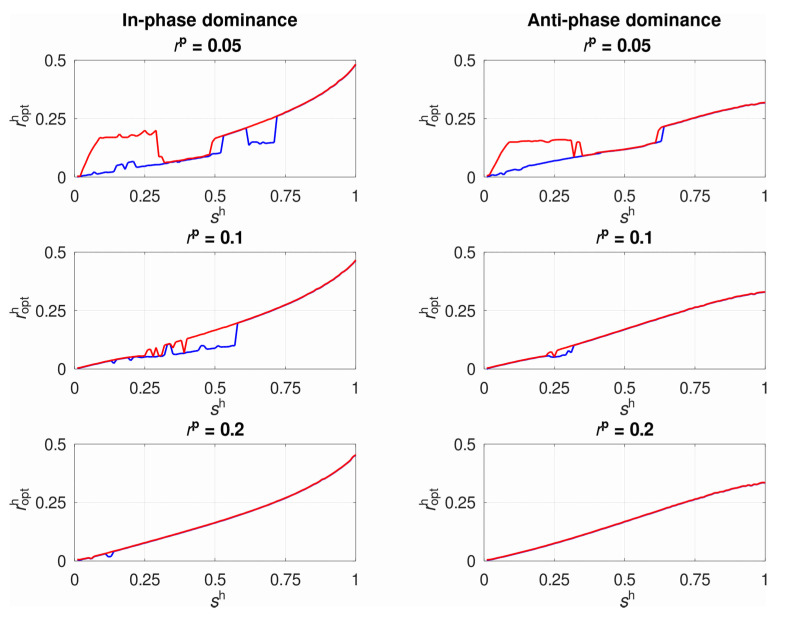
The effect of recombination rate in the parasite ( $r^{p}$ ) on the estimation of the optimal constant recombination rate in the host ( $r_{\mathrm{opt}}^{h}$ ). The colored curves show the lower (blue) and the upper (red) estimates. All simulations are conducted for the case of obligate parasitism (=1).

Additionally, we examined in detail the case of obligate parasitism, implying that the parasite survives only if it succeeds to infect ( $s^{p}=1$ ). This case seems to be of high biological importance. Moreover, as we mentioned above, higher values of $s^{p}$ considerably regularized the system’s dynamics, making it much easier to interpret. Here, the optimal constant recombination rate in the host ( $r_{\mathrm{opt}}^{h}$ ) appeared to be mostly determined by its selection intensity ( $s^{h}$ ): $r_{\mathrm{opt}}^{h}$ monotonously increased with $s^{h}$ . The recombination rate in the parasite ( $r^{p}$ ) had a minor effect on $r_{\mathrm{opt}}^{h}$ but strongly affected the complexity of the system’s dynamics, so that rather regular oscillations occurred mostly under moderate-to-high $r^{p}$ . As a result, the estimation of $r_{\mathrm{opt}}^{h}$ often failed under $r^{p}<0.2$ (Figure 1).

### 3.2. Selection for Plastic Recombination in the Host

Whenever the optimal constant recombination rate in the host was estimated with sufficient accuracy, it was compared with the two above-described forms of plastic, pathogen-inducible recombination: prevention strategy—with recombination rate increased proportionally to the risk of infection, and remediation strategy—with recombination rate increased upon the fact of infection.

**Figure 2:**
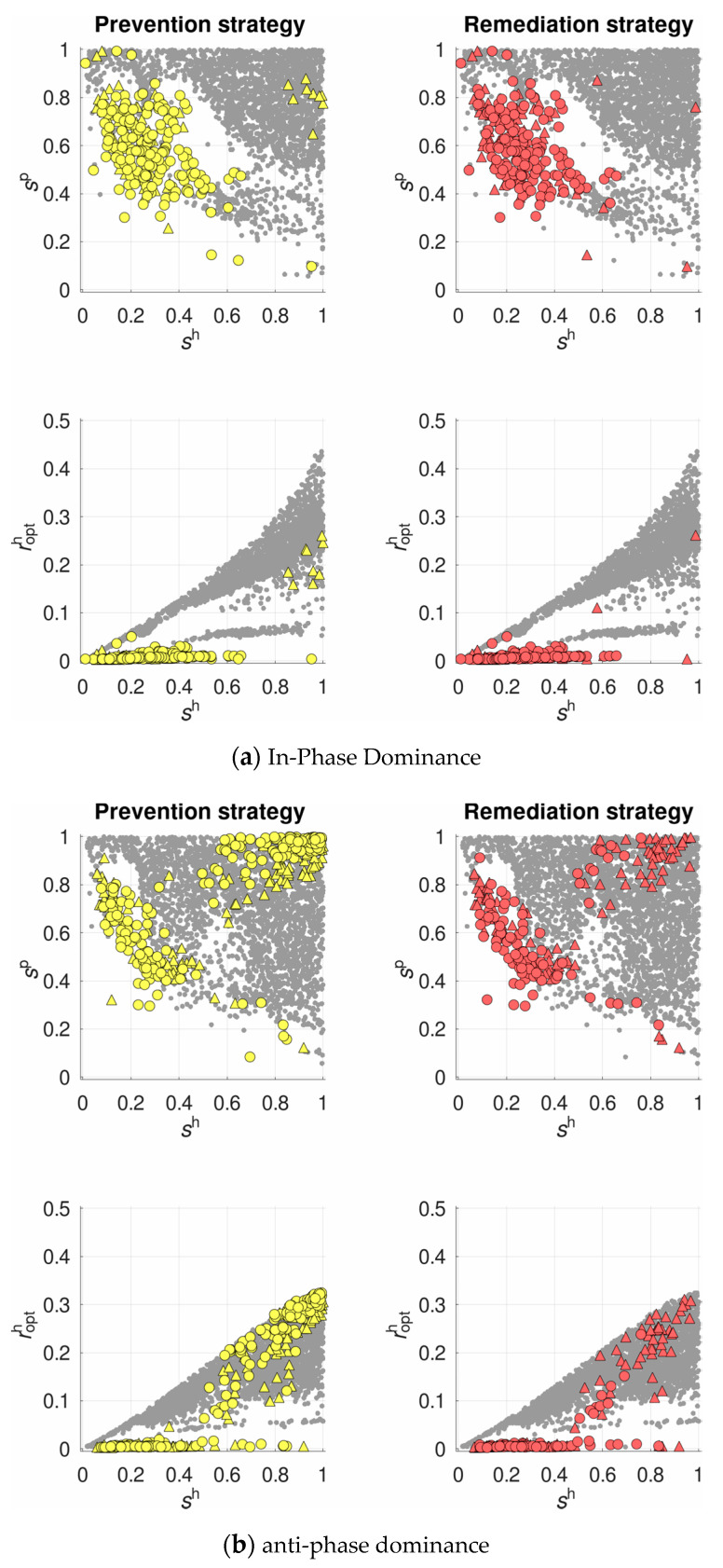
The evolutionary advantage of plastic recombination over non-zero optimal constant recombination, under in-phase (**a**) and anti-phase (**b**) dominance. The colored markers show regimes where the prevention (yellow) and the remediation (red) strategies are favored, either totally (circles) or partially (triangles).

We found plastic recombination is favored over non-zero optimal constant recombination quite infrequently, in ~9–11% of regimes. They were located in two quite distinguishable areas of the parameter space (Figure 2). The first area was presented by regimes with strong selection in both antagonists: $s^{h}>0.7$ , $s^{p}>0.7$ (a compact cluster of colored points at the top right corner of the coordinate plane $s^{h}$ – $s^{p}$ ). The overall selection in such regimes was moderate-to-strong: $s^{h}\cdot s^{p}>$ 0.5. Regimes of this type ensured a moderate-to-high recombination rate in the host: $r_{\mathrm{opt}}^{h}>0.1$ . The second area was presented by regimes with weak-to-moderate selection in the host and moderate-to-strong selection in the parasite: $s^{h}<0.6$ , $s^{p}>0.4$ (an arc-shaped cluster of colored points at the diagonal of the coordinate plane $s^{h}$ – $s^{p}$ ). At that, the overall selection was still relatively weak: $s^{h}\cdot s^{p}<$ 0.3. Such regimes ensured a low recombination rate in the host: $r_{\mathrm{opt}}^{h}<0.05$ . In general, plastic recombination was favored more often under anti-phase than in-phase dominance. Moreover, the prevention strategy was favored more often than the remediation one, especially under anti-phase dominance. Specifically, under in-phase dominance and strong selection, the prevention strategy was favored only partially, while the remediation strategy was not favored at all (Figure 2).

**Figure 3:**
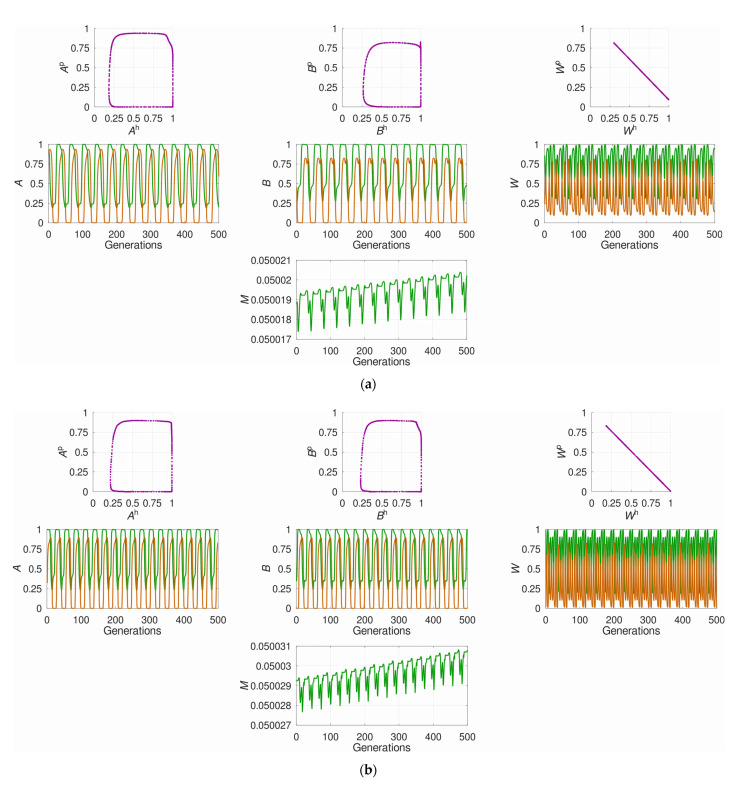

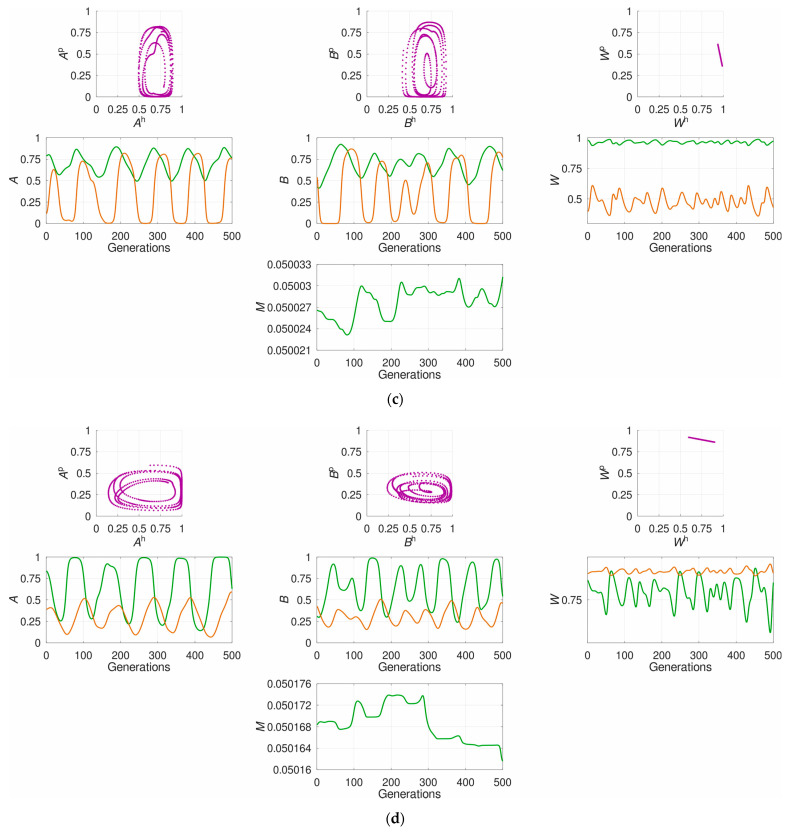
The system’s dynamics under different regimes favoring plastic recombination. The plots show the last 500 out of 10,000 generations of the competition between the optimal constant recombination and plastic recombination; yet, the pattern is qualitatively similar also for other time windows. The colored curves stand for the host (green) and the parasite (orange). *A*, *B* and *M* denote, respectively, the two interaction-mediating loci and the modifier locus while *W* denotes the population’s mean fitness. All examples stand for anti-phase dominance and prevention strategy: (**a**) A regime with strong overall selection: $s^{h}\approx 0.88$ , $s^{p}\approx 0.91$ . The optimal constant recombination in the host is high: $r_{\mathrm{opt}}^{h}\approx 0.25.$ The oscillations are fairly regular. The modifier allele for plastic recombination generally increases in frequency, again with fairly regular oscillations; (**b**) A regime with extremely strong overall selection: $s^{h}\approx 0.98$ , $s^{p}>0.99$ . The optimal constant recombination in the host is high: $r_{\mathrm{opt}}^{h}=0.32.$ The oscillations are regular. The modifier allele for plastic recombination generally increases in frequency, again with fairly regular oscillations; (**c**) A regime with weak overall selection due to weak selection in the host: $s^{h}\approx 0.14$ , $s^{p}\approx 0.70$ . The optimal constant recombination in the host is very low: $r_{\mathrm{opt}}^{h}<0.01.$ The oscillations are irregular. Although the modifier allele for plastic recombination generally increases in frequency, its oscillations are substantially irregular; (**d**) A regime with weak overall selection due to weak selection in the parasite: $s^{h}\approx 0.85$ , $s^{p}\approx 0.16$ . The optimal constant recombination in the host is very low: $r_{\mathrm{opt}}^{h}<0.01.$ The oscillations are irregular. Although the modifier allele for plastic recombination generally increases in frequency, its dynamics are considerably irregular; in certain time windows (like here), the decline of the modifier allele for plastic recombination may even temporally prevail.

Further analysis showed that the two above-described groups of regimes favoring plastic recombination (with moderate-to-strong and weak overall selection) considerably differed in the system’s dynamics. In the area of strong overall selection, the oscillations were highly regular, with a rectangular-like phase portrait for allele frequencies and a linear phase portrait for mean fitnesses (Figure 3a). Moreover, under extremely strong overall selection, the phase trajectories tended to become partially “punctuated” (Figure 3b). In contrast, in the area of weak overall selection, the oscillations were much more irregular, with spiral-like phase portraits. Such a pattern emerged regardless of which of the two selection intensities, either in the host or in the parasite, was weak (Figure 3c,d).

In the additionally examined case of obligate parasitism ( $s^{p}=1$ ), selection for plastic recombination was observed only under anti-phase dominance. Regimes favoring plastic recombination were characterized by strong selection in the host: $s^{h}>0.8$ . In total, their proportion reached ~20%.

**Figure 4:**
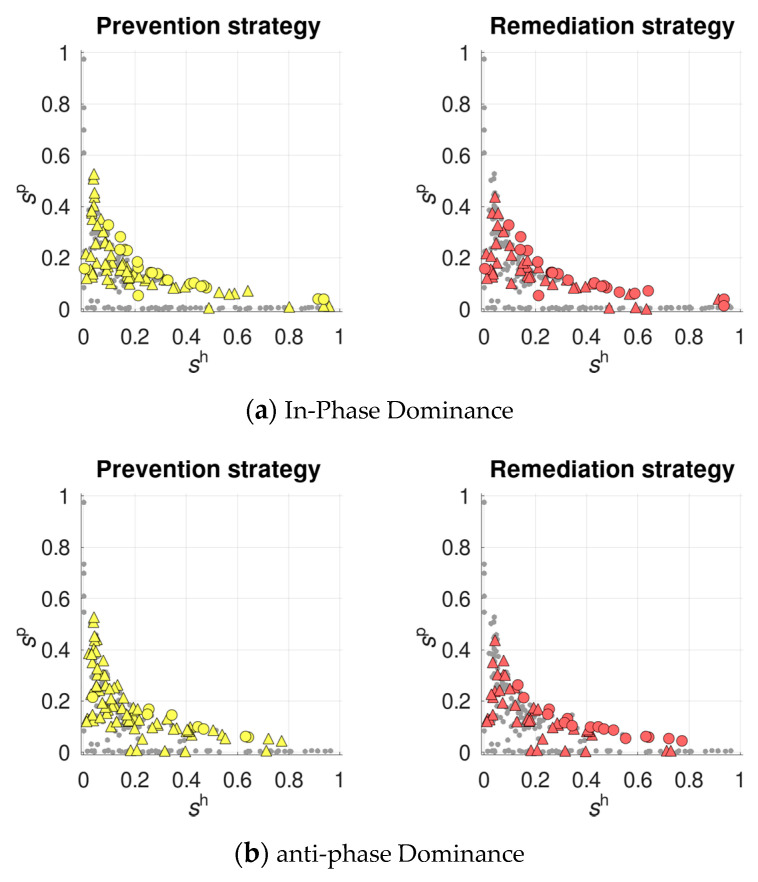
The evolutionary advantage of plastic recombination over zero optimal constant recombination, under in-phase (**a**) and anti-phase (**b**) dominance. The colored markers show regimes where the prevention (yellow) and the remediation (red) strategies are favored, either totally (circles) or partially (triangles).

As mentioned above, under sufficiently weak overall selection ( $s^{h}\cdot s^{p}<0.1$ ), any constant recombination in the host was rejected ( $r_{\mathrm{opt}}^{h}=0$ ). Remarkably, even in such conditions, plastic recombination was favored rather frequently: in ~34–37% of regimes. This happened even though it shifted the population’s mean recombination rate from zero, i.e., from the optimum in the class of constant strategies for the considered parameter combinations. Regimes favoring plastic recombination were characterized by the strongest (among those rejecting any constant recombination) overall selection. Moreover, they had a rather high recombination rate in the parasite: $r^{p}>0.2$ (yet, the latter constrain probably resulted from the earlier filtering, since higher values of $r^{p}$ tended to regularize the system’s dynamics). Again, the prevention strategy was favored easier than the remediation one—similar to regimes with non-zero optimal constant recombination (Figure 4).

**Figure 5:**
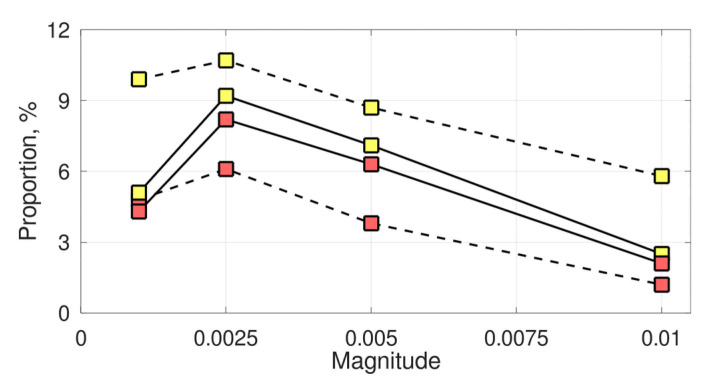
The effect of the magnitude of recombination plasticity on the proportion of regimes favoring plastic recombination. The line styles stand for in-phase (sold) and anti-phase (dashed) dominance, while the marker colors stand for prevention (yellow) and remediation (red) strategies.

The observed evolutionary advantage of plastic recombination substantially depended on the magnitude of recombination plasticity ( ∆r ), i.e., the difference in recombination rate between the infected and non-infected hosts. Specifically, plastic recombination was the most favored under ∆r=0.0025 , while both lower and higher magnitudes decreased the proportion of regimes favoring plastic recombination by 2–4 times, depending on strategy and dominance (Figure 5).

## 4. Discussion

The Red Queen hypothesis predicts that antagonistic interactions, like those between a host and its parasite, generate negative frequency-dependent selection on the interaction-mediating genes, which can give rise, at least under certain conditions, to indirect selection on recombination. Several empirical studies have indeed supported this assumption [30,31]. Moreover, the increased recombination seems to evolve easier within the clusters of infection-related genes [47,48,49]. Sometimes, such local induced-recombination hotspots occur in genomes with on average quite a low recombination rate, feeding the concept of so-called two-speed genome evolution [50].

Plastic, pathogen-inducible recombination might be viewed as another mechanism for differential, fine-tuned recombination evolution. Empirical studies, in general, confirm pathogens’ ability to increase the hosts’ recombination [36,37,38,39] (but see [51,52]). In the current study, we tested whether recombination’s sensitivity to pathogens could evolve as an adaptive strategy of the host. We examined the so-called “matching-phenotype” interaction—one of the standard types of interactions, implying that infection develops upon the complete phenotypic matching between the two antagonists (see [19] for a review of the interaction types). In the considered model, a certain recombination rate in the host was typically favored over all other, both lower and higher, rates. Sometimes, the system’s dynamics demonstrated bistability, i.e., two recombination rates appeared locally optimal. This phenomenon is well-known also for other types of recombination evolution models [53,54]. Anyway, plastic, pathogen-inducible recombination in the host under certain parameter combinations appeared even more favorable than the optimal (either globally or locally) constant recombination.

There exist several explanations for the evolutionary advantage of plastic recombination. The first, the so-called “abandon-ship” mechanism, appeals to the “selfish” benefits of the recombination modifier capable to adjust its linkage to the selected system [55,56]. The alternative, although non-exclusive mechanism claims that plastic recombination within the selected system helps protect advantageous allele combinations. Since here we intentionally excluded selfish benefits by assuming unlinked modifier locus, the observed evolutionary advantage of plastic recombination should be ascribed entirely to the benefits for the selected system.

Importantly, the population’s mean recombination rate under a plastic strategy may depart from the optimal constant recombination rate—even if this plastic strategy implies that the recombination rate varies around the optimum. Thus, a plastic strategy may be favored just due to a “better” (i.e., closer to the true optimum) mean recombination rate, rather than to the above-mentioned benefits of recombination-rate plasticity. To exclude such a trivial effect, we applied the “two-reference” test offered by Aggarwal et al. [56]. Specifically, we accomplished the “fringe” plastic strategy, implying recombination variation *around* the optimal rate $r_{\mathrm{opt}}^{h}$ (i.e., from $r_{\min}^{h}=r_{\mathrm{opt}}^{h}-∆r$ to $r_{\max}^{h}=r_{\mathrm{opt}}^{h}+∆r$ ), by two “one-sided” strategies: (1) with recombination varying *below* the optimum (i.e., from $r_{\min}^{h}=r_{\mathrm{opt}}^{h}-∆r$ to $r_{\max}^{h}=r_{\mathrm{opt}}^{h}$ ), and (2) with recombination varying *above* the optimum (i.e., from $r_{\min}^{h}=r_{\mathrm{opt}}^{h}$ to $r_{\max}^{h}=r_{\mathrm{opt}}^{h}+∆r$ ). The observed evolutionary advantage of a plastic strategy should be ascribed to the benefits of recombination plasticity per se only if both one-sided strategies, recombination-increasing and recombination-decreasing ones, are also favored over the optimal constant recombination. This criterion is very strict since each of the two one-sided strategies moves the population’s mean recombination rate from the optimum much stronger than the fringe strategy. Yet, the predominant proportion (~73–83%, depending on dominance) of regimes favoring plastic recombination succeeded to pass the two-referee test, proving the benefits of recombination plasticity.

Since the evolutionary advantage of a plastic strategy is determined by the trade-off between the benefits of recombination plasticity and the costs of departure from the optimal rate, it must be sensitive to the magnitude of recombination plasticity. In our simulations, plastic recombination was the most favored under an intermediate magnitude ( ∆r=0.0025 ). This suggests that under lower magnitudes, the benefits of recombination plasticity are weaker, while under higher magnitudes, the departure from the optimal rate becomes too costly.

Earlier, the evolutionary advantage of plastic recombination was theoretically demonstrated under forced oscillations, associated with the periodical abiotic environment, both for haploids [55] and diploids [40,41]. Usually, such abiotic fluctuations are fairly regular; however, even the model with stochastic, Markov-chain fluctuations led to qualitatively similar results [40]. Herein presented findings extend the conclusions of the previous studies to more complex auto-oscillations caused by antagonistic interactions. However, the evolutionary advantage of plastic recombination under antagonistic interactions was observed less frequently than in the above-mentioned models with forced oscillations: up to 11% of all regimes with regular oscillations, and up to 20% of regimes with obligate parasitism. Moreover, they required lower magnitudes of recombination plasticity. This discrepancy probably arises from considerably longer periods in the current study: even under the strongest overall selection, they exceeded 25 generations (see Figure 3b), whereas the cycles considered in [40,41] were up to 20 generations. Potentially, plastic recombination would evolve under a wider parameter range given further strengthening selection pressure, e.g., by assuming several generations of the parasite per generation of the host [57,58], similarity selection [59], or external abiotic selection [60].

The existence of two groups of regimes favoring plastic recombination (see Figure 2b) requires further in-depth analysis. Probably, in the area of low overall selection, where the system’s dynamics appeared much less regular, the complex oscillations can be presented as a superposition of several components with different periods. In this case, the evolution of recombination is likely determined by the longest-period component (“low-frequency filter”) [54,61]. Moreover, the peculiarities of the system’s dynamics probably explain a more frequent selection for prevention than remediation strategy. It turned out that the oscillations of mean fitness usually have a more complex profile than those of allele and genotype frequencies, with an about twice-higher frequency (see Figure 3b,c). Thus, the prevention strategy likely benefits from a finer “sensing” the whole population of the parasite.

Regimes where plastic recombination was favored over zero optimal constant recombination seem to be of special evolutionary importance. They suggest that recombination plasticity can serve as an evolutionary rescue for recombination, which otherwise would have disappeared from the host population. Notably, in such situations, plastic recombination is favored despite the above-discussed cost of moving the population’s mean recombination rate from the optimum. This finding expands our recent results showing that fitness-dependent recombination can save recombination from disappearing under purifying selection in mutation-selection models [62,63].

Several further directions are promising. First, in our model, we assumed only a diploid pathogen. With full dominance within the selected loci, the herein used matching-phenotype interaction becomes very similar to the matching-genotype interaction used in haploid models. Yet, an explicit comparison is desirable. Second, the considered pathogen-induced plasticity of recombination represents only one aspect of a wider phenomenon—condition dependence of recombination. The other aspect is whether and to which extent recombination per se and its plasticity to environmental stressors, including pathogens, are modulated by genotype fitness. To address this question, we need to model larger genetic systems, with at least three selected loci, which can provide the required variation in fitness across genotypes [41,62].

## Figures and Tables

**Table 1:** Interaction matrix: the two possible outcomes of the interaction as a function of the antagonists’ genotypes. Red and green cells stand, respectively, for infection (I) and resistance (R).

Parasite Genotype	Host Genotype								
	$A_{1}^{h}A_{1}^{h}B_{1}^{h}B_{1}^{h}$	$A_{1}^{h}A_{1}^{h}B_{1}^{h}B_{2}^{h}$	$A_{1}^{h}A_{1}^{h}B_{2}^{h}B_{2}^{h}$	$A_{1}^{h}A_{2}^{h}B_{1}^{h}B_{1}^{h}$	$A_{1}^{h}A_{2}^{h}B_{1}^{h}B_{2}^{h}$	$A_{1}^{h}A_{2}^{h}B_{2}^{h}B_{2}^{h}$	$A_{2}^{h}A_{2}^{h}B_{1}^{h}B_{1}^{h}$	$A_{2}^{h}A_{2}^{h}B_{1}^{h}B_{2}^{h}$	$A_{2}^{h}A_{2}^{h}B_{2}^{h}B_{2}^{h}$
In-Phase Dominance ( $A_{1}^{p}<A_{2}^{p};B_{1}^{p}<B_{2}^{p};A_{1}^{h}<A_{2}^{h};B_{1}^{h}<B_{2}^{h}$ )									
$A_{1}^{p}A_{1}^{p}B_{1}^{p}B_{1}^{p}$	I	R	R	R	R	R	R	R	R
$A_{1}^{p}A_{1}^{p}B_{1}^{p}B_{2}^{p}$	R	I	I	R	R	R	R	R	R
$A_{1}^{p}A_{1}^{p}B_{2}^{p}B_{2}^{p}$	R	I	I	R	R	R	R	R	R
$A_{1}^{p}A_{2}^{p}B_{1}^{p}B_{1}^{p}$	R	R	R	I	R	R	I	R	R
$A_{1}^{p}A_{2}^{p}B_{1}^{p}B_{2}^{p}$	R	R	R	R	I	I	R	I	I
$A_{1}^{p}A_{2}^{p}B_{2}^{p}B_{2}^{p}$	R	R	R	R	I	I	R	I	I
$A_{2}^{p}A_{2}^{p}B_{1}^{p}B_{1}^{p}$	R	R	R	I	R	R	I	R	R
$A_{2}^{p}A_{2}^{p}B_{1}^{p}B_{2}^{p}$	R	R	R	R	I	I	R	I	I
$A_{2}^{p}A_{2}^{p}B_{2}^{p}B_{2}^{p}$	R	R	R	R	I	I	R	I	I
Anti-Phase Dominance ( $A_{1}^{p}<A_{2}^{p};B_{1}^{p}<B_{2}^{p};A_{1}^{h}>A_{2}^{h};B_{1}^{h}>B_{2}^{h}$ )									
$A_{1}^{p}A_{1}^{p}B_{1}^{p}B_{1}^{p}$	I	I	R	I	I	R	R	R	R
$A_{1}^{p}A_{1}^{p}B_{1}^{p}B_{2}^{p}$	R	R	I	R	R	I	R	R	R
$A_{1}^{p}A_{1}^{p}B_{2}^{p}B_{2}^{p}$	R	R	I	R	R	I	R	R	R
$A_{1}^{p}A_{2}^{p}B_{1}^{p}B_{1}^{p}$	R	R	R	R	R	R	I	I	R
$A_{1}^{p}A_{2}^{p}B_{1}^{p}B_{2}^{p}$	R	R	R	R	R	R	R	R	I
$A_{1}^{p}A_{2}^{p}B_{2}^{p}B_{2}^{p}$	R	R	R	R	R	R	R	R	I
$A_{2}^{p}A_{2}^{p}B_{1}^{p}B_{1}^{p}$	R	R	R	R	R	R	I	I	R
$A_{2}^{p}A_{2}^{p}B_{1}^{p}B_{2}^{p}$	R	R	R	R	R	R	R	R	I
$A_{2}^{p}A_{2}^{p}B_{2}^{p}B_{2}^{p}$	R	R	R	R	R	R	R	R	I

**Table 2:** The relative effects of the examined parameters ( $s^{h}$ , $s^{p}$ , and $r^{p}$ ) and their combinations on the optimal constant recombination rate in the host ( $r_{\mathrm{opt}}^{h}$ ).

Parameters/Combinations	In-Phase Dominance (*n* = 2856)			Anti-Phase Dominance (*n* = 3307)		
	Model A	Model B	Model C	Model A	Model B	Model C
Selection Intensity in the Host ( $s^{h}$ )	0.787	-	0.137	0.810	-	0.238
Selection Intensity in the Parasite ( $s^{p}$ )	0.425	-	−0.040	0.479	-	0.029
Recombination Rate in the Parasite ( $r^{p}$ )	0.023	0.022	0.037	0.032	0.040	0.049
Overall Selection Intensity ( $s^{h}\cdot s^{p}$ )	-	0.974	0.889	-	0.970	0.775
*R*^2^-Adjusted	0.891	0.949	0.961	0.901	0.942	0.962

## Data Availability

The codes used for the simulations are available in Supplement S1.

## Supplementary Materials

The following are available online at https://www.mdpi.com/2076-0817/10/7/898/s1, File S1: The codes used for the simulations, Figure S1 The effects of the examined parameters ( $s^{h}$ , $s^{p}$ , and $r^{p}$ ) on the system’s dynamics.
