# Erratum: Rybnikov et al. Selection for Plastic, Pathogen-Inducible Recombination in a Red Queen Model with Diploid Antagonists. *Pathogens* 2021, *10*, 898

**DOI:** 10.3390/pathogens10111460

**Published:** 2021-11-11

**Authors:** Sviatoslav Rybnikov, Zeev Frenkel, Abraham B. Korol, Tzion Fahima

**Affiliations:** 1 Institute of Evolution, University of Haifa, 199 Abba-Hushi Avenue, Haifa 3498838, Israel; sviatoslav.rybnikov@gmail.com (S.R.); zvfrenkel@gmail.com (Z.F.); 2 Department of Evolutionary and Environmental Biology, University of Haifa, 199 Abba-Hushi Avenue, Haifa 3498838, Israel

In the original article, there was a mistake published in Figure 3 [1]. Figure 3c,d in the published version are identical: instead of inserting Figure 3d, we by mistake repeated Figure 3c. In the legend to Figure 3 (line 4), change “for the modifier locus” to “the modifier locus”. The correct Figure 3d appears below. 

In the titles to Figure 2b and Figure 4b, “Antiphase” changes to ‘anti-phase” (the hyphen is used throughout the text).

The last keyword should be “model” instead of “nodal”. 

The authors and editorial office would like to apologize for any inconvenience caused to the readers by these changes and state that the scientific conclusions are unaffected. The original article has been updated. 

## Figures and Tables

**Figure 3:**
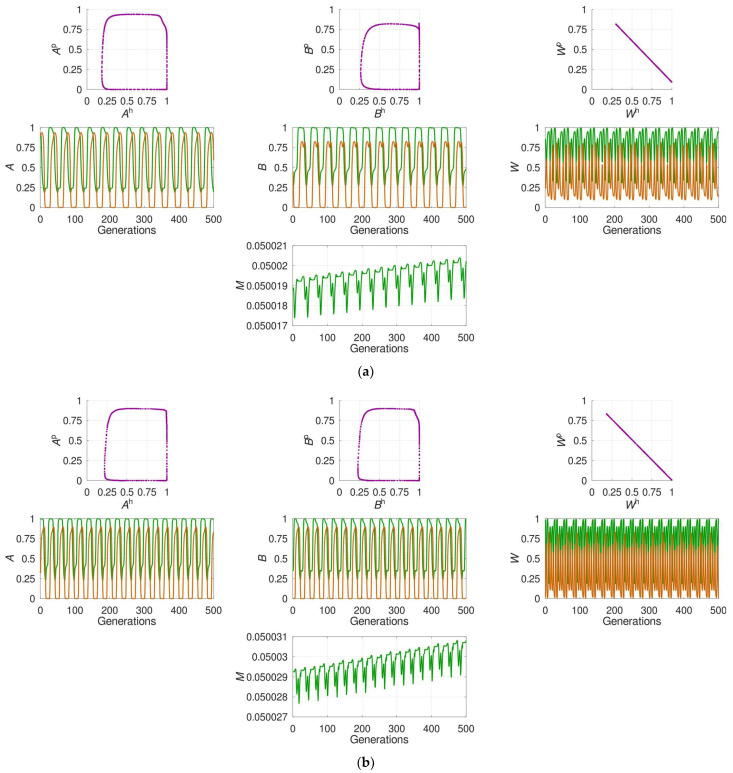

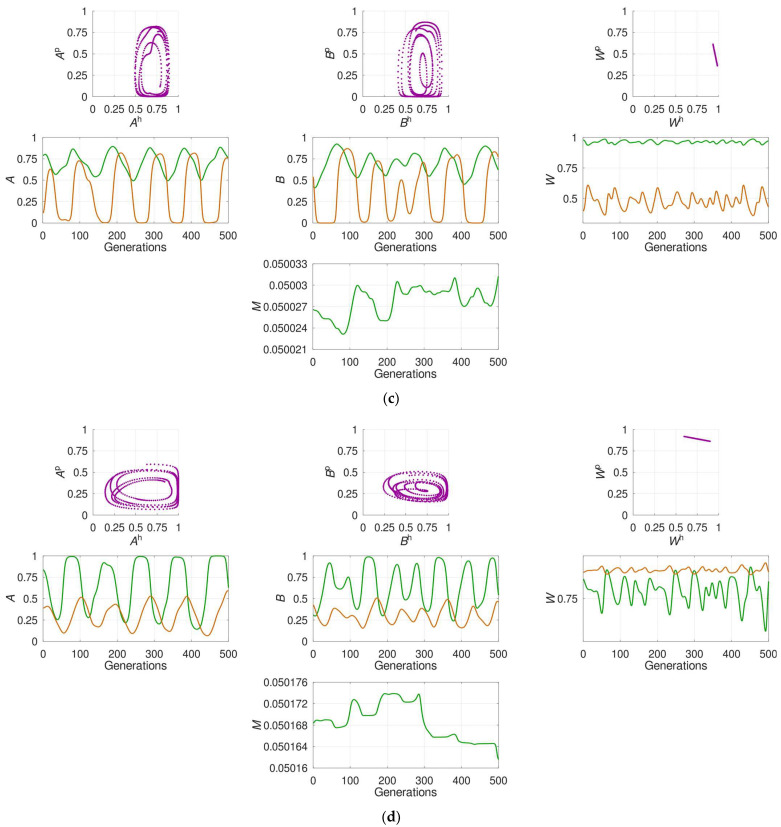
The system’s dynamics under different regimes favoring plastic recombination. The plots show the last 500 out of 10,000 generations of the competition between the optimal constant recombination and plastic recombination; yet, the pattern is qualitatively similar also for other time windows. The colored curves stand for the host (green) and the parasite (orange). *A*, *B* and *M* denote, respectively, the two interaction-mediating loci and the modifier locus while *W* denotes the population’s mean fitness. All examples stand for anti-phase dominance and prevention strategy: (**a**) A regime with strong overall selection: $s^{h}\approx 0.88$, $s^{p}\approx 0.91$. The optimal constant recombination in the host is high: $r_{\mathrm{opt}}^{h}\approx 0.25.$ The oscillations are fairly regular. The modifier allele for plastic recombination generally increases in frequency, again with fairly regular oscillations; (**b**) A regime with extremely strong overall selection: $s^{h}\approx 0.98$, $s^{p}>0.99$. The optimal constant recombination in the host is high: $r_{\mathrm{opt}}^{h}=0.32.$ The oscillations are regular. The modifier allele for plastic recombination generally increases in frequency, again with fairly regular oscillations; (**c**) A regime with weak overall selection due to weak selection in the host: $s^{h}\approx 0.14$, $s^{p}\approx 0.70$. The optimal constant recombination in the host is very low: $r_{\mathrm{opt}}^{h}<0.01.$ The oscillations are irregular. Although the modifier allele for plastic recombination generally increases in frequency, its oscillations are substantially irregular; (**d**) A regime with weak overall selection due to weak selection in the parasite: $s^{h}\approx 0.85$, $s^{p}\approx 0.16$. The optimal constant recombination in the host is very low: $r_{\mathrm{opt}}^{h}<0.01.$ The oscillations are irregular. Although the modifier allele for plastic recombination generally increases in frequency, its dynamics are considerably irregular; in certain time windows (like here), the decline of the modifier allele for plastic recombination may even temporally prevail.
