# Supplementary material for: Selection for Plastic, Pathogen-Inducible Recombination in a Red Queen Model with Diploid Antagonists
Source: Pathogens. 2021 Jul 15;10(7):898. doi: 10.3390/pathogens10070898 (PMC8308896; doi:10.3390/pathogens10070898)
Supplement: Supplementary file 1 [file pathogens-10-00898-s001.zip › pathogens-1220334 - Figure S1.pdf]

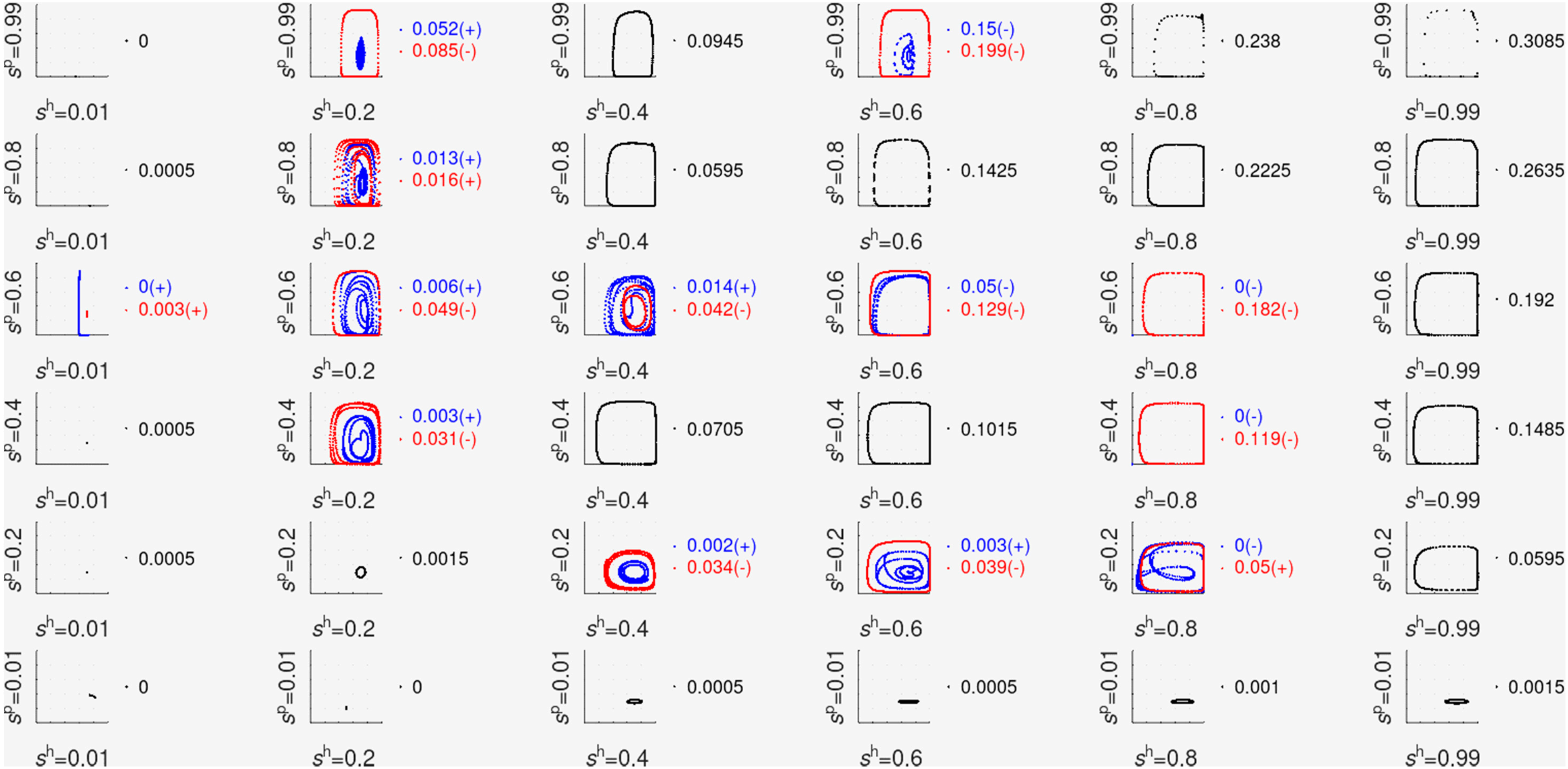

s^p=0.99
s^h=0.01
0
s^h=0.2
0.052(+)
0.085(-)
s^h=0.4
0.0945
s^h=0.6
0.15(-)
0.199(-)
s^h=0.8
0.238
s^h=0.99
0.3085
s^p=0.8
s^h=0.01
0.0005
s^h=0.2
0.013(+)
0.016(+)
s^h=0.4
0.0595
s^h=0.6
0.1425
s^h=0.8
0.2225
s^h=0.99
0.2635
s^p=0.6
s^h=0.01
0(+)
0.003(+)
s^h=0.2
0.006(+)
0.049(-)
s^h=0.4
0.014(+)
0.042(-)
s^h=0.6
0.05(-)
0.129(-)
s^h=0.8
0(-)
0.182(-)
s^h=0.99
0.192
s^p=0.4
s^h=0.01
0.0005
s^h=0.2
0.003(+)
0.031(-)
s^h=0.4
0.0705
s^h=0.6
0.1015
s^h=0.8
0(-)
0.119(-)
s^h=0.99
0.1485
s^p=0.2
s^h=0.01
0.0005
s^h=0.2
0.0015
s^h=0.4
0.002(+)
0.034(-)
s^h=0.6
0.003(+)
0.039(-)
s^h=0.8
0(-)
0.05(+)
s^h=0.99
0.0595
s^p=0.01
s^h=0.01
0
s^h=0.2
0
s^h=0.4
0.0005
s^h=0.6
0.0005
s^h=0.8
0.001
s^h=0.99
0.0015

$r^p = 0.05$

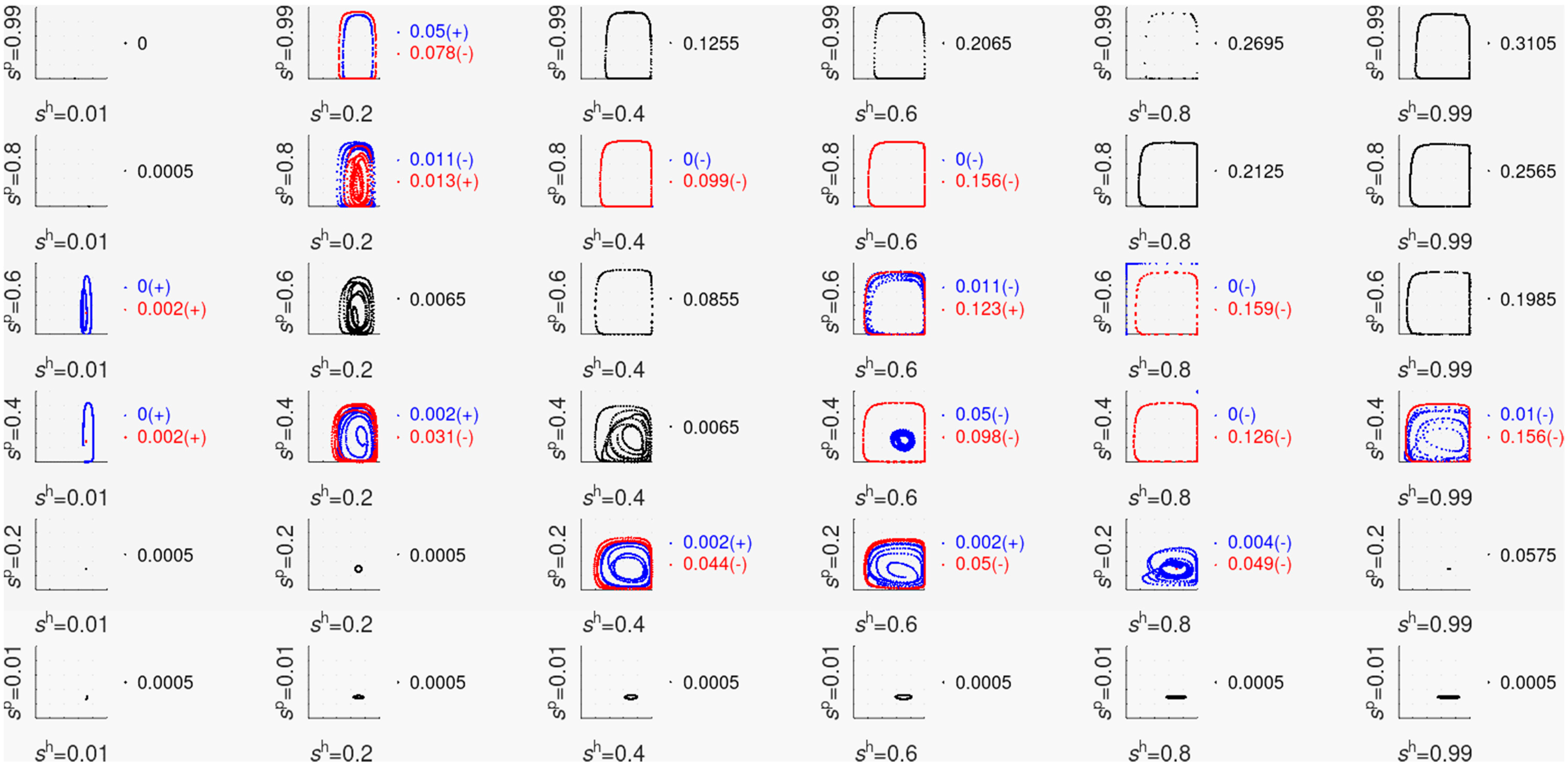

sp=0.99
0
0.05(+)
0.078(-)
0.1255
0.2065
0.2695
0.3105
sp=0.8
0.0005
0.011(-)
0.013(+)
0(-)
0.099(-)
0(-)
0.156(-)
0.2125
0.2565
sp=0.6
0(+)
0.002(+)
0.0065
0.0855
0.011(-)
0.123(+)
0(-)
0.159(-)
0.1985
sp=0.4
0(+)
0.002(+)
0.002(+)
0.031(-)
0.0065
0.05(-)
0.098(-)
0(-)
0.126(-)
0.01(-)
0.156(-)
sp=0.2
0.0005
0.0005
0.002(+)
0.044(-)
0.002(+)
0.05(-)
0.004(-)
0.049(-)
0.0575
sp=0.01
0.0005
0.0005
0.0005
0.0005
0.0005
0.0005
sh=0.01
sh=0.2
sh=0.4
sh=0.6
sh=0.8
sh=0.99

$r^{p} = 0.1$

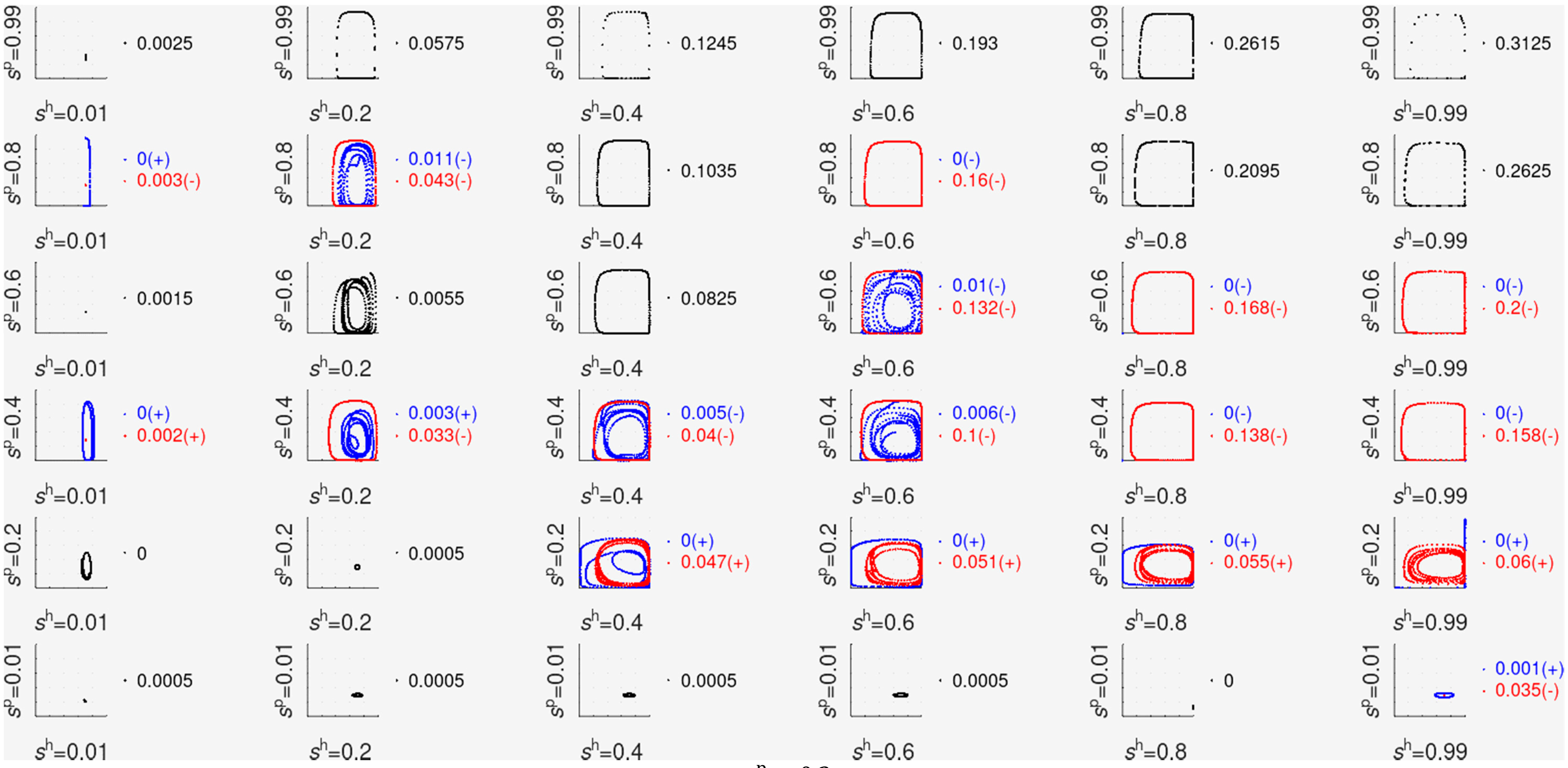

$r^{\mathrm{p}} = 0.2$

Figure S2. The effects of the examined parameters (s^{h}, s^{p}, and r^{p}) on the system's dynamics. The plots show the last 500 out of 10,000 generations of the 'burn-in' period (with the monomorphic recombination modifier in the host). The colored trajectories stand for the dynamics with lower (blue) and the upper (red) estimates of the optimal cantante recombination in the host ($r^{\mathrm{h}}_{\mathrm{opt}}$); whenever the two estimates sufficiently converged ($\varepsilon \leq 0.001$), only one trajectory is shown (black). The signs near the estimates show the outcome of their direct

competition: (*i*) one rate (+) is favored over the other (–); (*ii*) both rates spread from leading to polymorphism (+/+); (*iii*) both rate spread from abundance leading to bistability (–/–).
